# New Jersey State Dental Society

**Published:** 1889-12

**Authors:** 


### NEW JERSEY STATE DENTAL SOCIETY.—(*Continued.*)

### DISCUSSION OF DR. GEORGE S. ALLAN'S PAPER ON "PYORRHŒA ALVEOLARIS.

*Dr. James Truman.*—Mr. Chairman, I am placed in rather an embarrassing position, in having to talk to an audience composed largely of gentlemen who did not hear Dr. Allan's essay this morning, and many of whom probably have come to listen to the paper that will succeed my remarks; however, I will endeavor to condense, as much as possible, what I have to say in answer to Dr. Allan's paper.

The first point that seemed to me to require attention was his statement that the investigations of Dr. Black, of Illinois, have given us all that we really know in regard to pyorrhœa alveolaris. Now, as this question has occupied the professional mind for the last twenty or twenty-five years, I do not think the statement can be wholly relied upon as true. No doubt, Dr. Allan thinks it is true. I am not willing to count myself second to any one in appreciation of Dr. Black's histological work; but long preceding his investigations, I think, the profession quite thoroughly understood the nature of this pathological condition. Twenty years ago I spent a great deal of time in the investigation of this subject, following the lead of the French workers, especially Dr. Magitot; but the results of treatment were not satisfactory.

Dr. Allan stated this morning that the loss of teeth from pyorrhœa alveolaris was greater, probably, than from all other causes combined, if I understood him correctly. That certainly is a mistake. It depends largely upon his definition of pyorrhœa alveolaris. Allowing the largest latitude for that definition, taking in all the senile teeth, and including all those that have suffered absorption through the action of tartar, as well as those that can be justly said to be destroyed by this disease, and we would not have a number that would at all compare, in my judgment, with the number destroyed by caries. But has he any right to take in senile teeth, and the teeth that have been destroyed by tartar? I contend that those teeth that have been injured by deposits, or lost in old age, where absorption has taken place, cannot properly be included; their loss is due to an entirely different action. Also those teeth that have been lost or loosened by salivation are, in my view, to be thrown out entirely; and we should then include only those that are affected by the pathological condition which we term pyorrhœa alveolaris.

The essayist took the position, if I understood him, that the presence of pus does not represent the disease. Now, what is pus? If I understand at all the origin of pus, and perhaps I do not, it is this: irritation precedes what we call inflammation, and inflammation produces what we term pus, from two processes: first, emigration of the so-called white blood-corpuscles; and, secondly, the breaking down of tissue and the retrogade metamorphosis of the tissue to its original protoplasmic elements. Is it possible to have inflammation without one or both of these processes going on? I certainly contend that it is not. The moment you have irritation you have an enlargement of the blood-vessels and a congested condition, and when that occurs, you have the emigration of the white blood-corpuscles, and eventually a breaking down of the tissue and the retrograde metamorphosis that I spoke of. Can we have inflammation, such as we understand by pyorrhœa, without pus? Certainly it does not appear to me to be so.

The essayist further stated that the name was a misnomer. That is, in one sense, true. But some words change their meaning. All words represent an idea, and that idea may change in the course of time. When the French writers named this disease pyorrhœa alveolaris, it meant to them precisely what the term conveyed,—pus originating from the alveolus, the bone surrounding the teeth. In the course of time that idea has changed, but the name remains; and I think it is well to continue it. If Dr. Allan had been a teacher as long as I have, probably he would understand how difficult it is to go before a class of young men and talk on subjects, and have to enter into a definition of the different terms, which have been given by various individuals when they had found those that represented their ideas, little thinking that in the course of time these will change, and their terms may no longer represent these pathological conditions. As a matter of fact, is this name entirely wrong? Dr. Black, whose work in this direction we all fully appreciate, has shown us most clearly that the periosteum is made up of inelastic fibers, that extend from the cement on the one hand to the bone on the other without break; and if inflammation occurs in the periosteum, it is necessarily carried to the cement on the one side and to the bone on the other. Therefore is it wrong to call this disease pyorrhœa alveolaris? I do not think it is. Nine times out of ten, the essayist stated, tartar was the cause of the disease. Now, if nine times out of ten tartar is the cause of this disease, we ought to have it in all cases where tartar is found. He acknowledged that ordinary salivary calculus was not the cause of this disease; but the tartar which he found upon the teeth or roots he called serumal tartar. He has taken up the old, old song of serumal tartar, and affirms dogmatically that it is serumal tartar. I have yet to learn of a single investigation that has been made to demonstrate the existence of this characteristic tartar. What is it? If I understand it, it means a calcareous deposit from the serum of the blood. We all know very well that there is scarcely an organ in the body that may not receive such deposits; but we have no evidence that this serumal tartar, so-called, comes from the serum of the blood. May it not come from pus? because pus can deposit calcareous particles as well as any fluid of the blood. As long as we do not know that it does come from the serum of the blood we have as much right to affirm—as this gentleman has to assume the contrary—that it is derived, as ordinary tartar, from the saliva.

Now, what is the genesis or origin of this pathological condition? Does it originate from tartar? Not if I understand it. Does it originate from the roughness that Dr. Allan spoke of, at the gingival border of the teeth? Possibly; but where does that roughness come from? When you take a patient in hand, and that patient states to you that in the morning when he brushes his teeth the blood will ooze from the gum, "that his teeth bleed," you know, and we all know, what that condition is. Here and there a tooth will present a bright red line at the border-line of the gum. The moment that is touched blood will ooze from it, by the disturbance of the capillaries at that point. That is the beginning. And if you take it in hand at that stage you can stop pyorrhœa alveolaris. It has nothing to do with tartar. It may come from constitutional disturbances; it may come from some form of nephritis, or a long siege of sickness. What then follows necessarily after this? Immediately succeeding we have a development of micro-organic life. That I demonstrated twenty years ago. On examination of these cases I found large quantities of bacteria throughout the broken-down tissue. I soon learned that they had something to do, although I did not then appreciate the full extent of their action, with all these inflammatory conditions; and you dentists, every one of you, can and do make those conditions in the mouths of your patients by careless work. When you place a rubber dam, or a clamp, or ligatures upon teeth, you produce irritation, and the patient will complain. You take off the instrument, and you allow the patient to go away without any treatment whatever. In fortyeight hours there will be a development of micro-organisms, and pain will result, and irritation at the neck of the tooth; and if it is not stopped at that time, it may go on until this pathological condition which we call pyorrhœa alveolaris appears.

What is the treatment? First, I hold that no dentist should put a rubber dam in the mouth, or a clamp on the teeth, or do anything of that kind that is liable to raise inflammation at the necks of the teeth, without applying an antiseptic. For this purpose I know nothing better than sulphate of quinia, mixed into a paste,—not because it is the best germicide, but because it is more lasting than other agents.

This disease has its origin in inflammation of the periosteum, or pericementum, of the roots of the teeth. There is no question about that. I am satisfied, by the investigations of others, that I have always been right in that respect, and I have been teaching it for many years. It naturally follows, therefore, that if we are to treat the teeth properly we must direct our attention to the micro-organic life first, and not to the tartar, which is secondary. Has Dr. Allan ever found his so-called tartar below the line of healthy pericementum? I cannot say that I ever did. The tartar comes in afterwards; first, disturbance of the pericementum, then the deposit of tartar. Remove that tartar and what is the result? Always a necrosed tissue. I have never succeeded in building up the periosteum beyond that line. Dr. Atkinson asserts that he can build the periosteum up to the gingival border of a tooth. I never have been able to do it. If there is tartar there, it should be removed, but it is not the original cause of the trouble; and Dr. Allan himself says that the roots of teeth that have been lost through this pathological condition are shiny and without periosteum. If that is the case, how is he going to do anything with it? The use of mechanical instruments should be secondary. I have treated successfully many cases of pyorrhœa alveolaris, and rarely have I used an instrument. I do not find so much of that tartar as some seem to do. I find it on senile teeth; but that is not pyorrhœa. I do not think any man here ever saw a case of pyorrhœa alveolaris on the lingual surface of the inferior central incisors. You find it on the anterior surfaces but not on the posterior; because the tartar is there to protect the lingual surface. Where tartar is there cannot arise—does not arise—this pathological condition, in my judgment.

The treatment is necessarily very much in accordance with that stated by Dr. Allan. I do not differ with him materially upon the subject generally. The points of variance I have endeavored to bring before you. In the treatment it is necessary first to remove any foreign body that may be present, whether food or other deposits; then inject into the pocket peroxide of hydrogen. I follow that up with sulphuric acid. I was very glad to find that Dr. Allan had been using altogether the commercial sulphuric acid. I long ago abandoned the use of aromatic sulphuric acid, as not being adapted to our purpose. I think we owe the introduction of this agent to Dr. Atkinson. I have used it for many years in this particular work. Magitot recommended chromic acid. I never had any satisfaction in its use, nor any of those more powerful agents. I use the ordinary commercial sulphuric acid, but stronger than Dr. Allan uses it; a twenty-five-per-cent. solution, and sometimes even stronger than that; but I do not allow it to remain on long. I apply it with a sharp stick around the teeth. It, of course, turns the parts of a dark color. I allow it to remain two or three minutes; not more; just sufficient to burn out the dead tissue; then immediately apply bicarbonate of soda, which brings away every portion of the dead matter. After a little time has elapsed I wash it out with warm water; then apply sulphate of quinia. I have used this for years successfully. If you want to know the philosophy of it, look into the materia medica books, and you will find it described. The sulphate of quinia will remain there longer than any other antiseptic that I know of. If the pain returns in the course of a few days, I would, of course, repeat the sulphuric acid treatment, and after that continue the antiseptic treatment. Then, after the parts have become perfectly healthy, you must use an antiseptic wash to keep them in proper condition. If the pocket remains, there will be a return of the disease, in spite of all your efforts, if you do not take necessary precautions to obliterate it.

This mode of treatment is not original with me, except the bicarbonate of soda and quinia. I have used these for several years with the most decided success and the greatest satisfaction.

*Dr. Sudduth.*—This is a subject of great interest to the profession, and, if time permitted, I would like to go into it fully. I can heartily second what Dr. Truman has said regarding the relation of serumal tartar to this disease, and the deposit found on the teeth being secondary to the disease. The initial phase of the disease we do not know. No man has ever been able to tell what is the cause of pyorrhœa alveolaris. There is a catarrhal process; but what causes that catarrhal process to be set up has not been as yet solved. The deposit on the roots of the teeth is evidently a result of the catarrhal process. First there is irritation; then follow microorganisms; and they in turn become a source of irritation, but their direct connection with the disease has never been determined.

The treatment that has been advocated—the use of aromatic sulphuric acid or commercial sulphuric acid—is good; but you should follow it up with some stimulating antiseptic. About a year ago I presented to the profession some tablets of silico-fluoride of soda, made after a formula of Mulford, of Philadelphia. It is coming to be used in surgical practice, especially in ophthalmic cases, and has almost taken the place of boric acid in operations on the eye. It is not poisonous. I mentioned this remedy before the Pennsylvania State Society a year ago last June. No one has taken it up, or, so far as I know, said anything about it. The object in bringing these remedies before the profession is to have them tested. This summer I have again distributed samples to members of the profession, hoping they would try them and report the results of their use in this treatment.

*Dr. Rhein.*—Mr. President, I want to speak of one point in Dr. Allan's paper; that is, the opinion he expresses that pyorrhœa alveolaris, as we see it, is never due to any constitutional affection, as I understand him. The very worst cases of pyorrhœa that have ever come under my notice have been cases absolutely devoid of tartar, either of the so-called serumal kind or any other. Speaking after considerable experience in the treatment of the disease, the opinion that I have come to hold is that where pyorrhœa is the result of some old constitutional trouble, as we frequently find it,—and we frequently find it in those chronic forms, some cases running for ten or fifteen years,—it results from some febrile disturbance, or some great disorder of the general system, which was cured, but left that condition of pyorrhœa, which became chronic. Those cases look the worst when they come to us; but they are always curable; and it is cases of this kind that have misled a large number of the profession to the belief that every case of pyorrhœa is curable. There are certain cases, which, to my mind, are not curable; at least I should be delighted to see them cured by any one. The great difficulty in coming to a determination in regard to this matter is that the majority of us make no effort at a differential diagnosis when we see a case for the first time. Our first duty should be to assure ourselves that the patient has existing in his body no pathological condition; and to that end I always insist upon making a very careful examination of every vital organ, including a thorough examination of the urine. The urine is really one of the most important means by which we can come to an opinion on the subject.

But no conclusion should be arrived at from a single examination; it should be taken at different intervals. By an examination of that kind we may find, in people who are subject to pyorrhœa and who are advanced in life, a sluggishness of the circulatory system which is sufficient to produce this result. In numerous cases where an examination has shown symptoms of Bright's disease, or phthisis, I have found that all the treatment and hygienic care that could be given would not prevent the disease returning in certain cases that were beyond medical treatment, as acknowledged by the best professional men that we have in New York City. I will give a practical case, showing that the disease is due to some pathological condition existing at the time.

A few months ago a gentleman came to me from out of town; his mouth was in very bad condition; pressure upon any tooth would cause a large exudation of pus; the gum around the gingival margins was whitened; there was a general hypertrophy of the tissues; and he expressed himself as utterly devoid of any hope of retaining those teeth in position. He was a young man, about thirty-five years of age. He had been with the first Stanley expedition through Africa, and in that expedition he had contracted that terrific fever which they have there; and he told me he had taken as much as eighty to one hundred grains of quinine a day. After an examination of his mouth, I could find no other cause for the condition of things than his African experience. I made a most careful examination in his case. I had the examination made by a specialist, who is capable of making both physical and urinal examinations; and his system was found to be otherwise in a perfectly healthy condition. That man's case surrendered to treatment, and his mouth is now in a healthy condition. Had I found the condition of Bright's disease in his system, as he imagined, I should never have given a good prognosis. I gave such a prognosis at the start, and his case at the present day bears me out in my judgment of the case.

*Dr. Allan.*—I do not know that I have anything further to say on the question, except it be in regard to Dr. Black's papers upon the subject. If there is anything in our literature which equals them in thoroughness of detail I have not been able to put my hand upon them. I am quite certain that those papers published in the "American System of Dentistry" are the best compendium of this subject that we have.

In regard to the number of teeth lost through this disease I still think I am right, according to the figures and data that I have been able to obtain, notwithstanding Dr. Truman's assertions to the contrary. I took great pains to hunt up statistics, and, I think, taking the definition of this trouble as an inflammation, or lesion of the peridental membrane, having its origin around the necks of the teeth, my statement is not so far from correct; but I was careful to state that it was only an approximate estimate, that the number of teeth lost through this disease might be equal to those lost through caries. Dr. Truman, I think, must have erred in his judgment or memory, as regards the formation of pus. It is not an absolutely necessary sequence or result of inflammation. Many inflammations exist in the body, in joints, and even near the surface, —even violent inflammations,—without the formation of pus, so long as micro-organisms are not present. Therefore, if the mucous membranes are maintained intact, there is no necessity that there should be any formation of pus in those places. Clinical examinations of cases, where tartar is present and the gums tumefied, have frequently shown them to be devoid of any indications of pus. I think I am right in saying that pus is not a necessary result of inflammation.

Dr. Truman speaks of my remark that, after dead teeth had been extracted, where the peridental membrane had been destroyed, and its connection with the circulation cut off, they were found to be, from the apical end to the gingival border, polished and clean. So I did; but I did not say it was so if the tartar had been left upon them. The supposition was that it had been removed. I spoke of that case to show the manner in which destruction of the peridental membrane takes place when the circulation had been cut off both from the apical and gingival borders.

I still hold to the point which I wish to make,—that a certain amount of roughness at the gingival border or neck of the tooth—as Dr. Sudduth suggested indirectly, and not with any intention of saying I had put it in my paper, but which I did have in my paper—may possibly be the occasion of the deposit of tartar; and that if the neck of the tooth is kept clean and polished, no tartar would form there, and we would not have pyorrhœa alveolaris in any of its forms. I am quite certain that, where the disease is so perfectly amenable to treatment,—following this line of thought,—and where we have perfect cures, such as I have frequently had without a particle of medication, but simply by mechanically removing the tartar, that the more this point is thought over and studied the more the theory will be adopted that pyorrhœa alveolaris has its origin in a deposit on the necks of the teeth from the gingiva. Whether the deposit is serumic in origin or not, I do not know. I do not think there is any indication whatever that it comes from the saliva. It is deposited under the margin of the gum, away from the openings of the salivary ducts, where the saliva has almost nothing to do with it. Whether it comes from the serum of the blood I do not know; but all its physical qualities are usually totally different from that of salivary calculus in every way. Therefore I think there is no ground for saying it comes from the saliva; and we are necessarily thrown back to another origin. If you can think of any other origin than the one Dr. Black has suggested, it is more than I can.

### DISCUSSION ON DR. ELLIOTT'S PAPER, ENTITLED "LIGHT TO THE GENTILES."

*Dr. W. H. Atkinson.*—I hesitated to open the discussion of this paper, although a somewhat intimate acquaintance with the author gave me a certain insight as to what I might prepare for,—satisfaction at the general trend of the doctrine pervading the paper, which is based on a knowledge that is not as yet broadspread among the gentiles, and not well understood even by those who claim to be light-bearers to these people who sit in darkness.

The two special prerequisites for successful teaching are ability in the teachers and capacity in and willingness of the learners faithfully to pursue the studies projected. A notable example of unwillingness to take the requisite labor of study to comprehend molecular phases was brought to my attention after hearing one of the best papers—if not the best—I have ever listened to from this author's pen. It was read before one of our dental societies, and a prominent member remarked, "Two more papers such as that will put this society in its grave," and this without dissent from those who heard the statement. Subsequently I said to Dr. Elliott, "Be patient, pursue work in the line of your present investigations, and the time will come when they will gladly hear you in even more profound disquisitions than the beautiful elaboration just displayed." That paper was upon carbon and its combinations. I endorse the commendation of this society for its zeal in pushing investigations, with a satisfaction akin to the zeal expressed by Dr. Elliott in accepting the invitation to prepare a paper. And I take occasion to notice this evidence of the fulfilment of the words of encouragement spoken to him on the occasion referred to, and also to congratulate the management and this society on seeking and interestedly listening to papers of this character.

Without a knowledge of how prime elements become proximate elements, of which tissues, organs, and systems are built, we shall be utterly at a loss to comprehend the functions of production, nutrition, growth, and decay; of which there are two marked varieties,—the decay of molecular metamorphosis, in which prime elements are used up in nourishing functioning bodies, and the retrogressive changes in the various forms of disease by which structures are destroyed beyond the power of unaided repair. The first is the result of the running out of the line of longevity of the tissue involved; and in every instance where uplastic pabulum is at hand, the tissue is maintained in full type and vigor—is physiological. The second is where retrogressive nutrient metamorphoses take place from deficiency or surcharge of pabulum, or pabulum with a cacoplastic tendency; and this gives the basis of pathology, demanding therapeusis to bring about favorable results.

We might go on with beautiful, gratuitous statements of the views we take of the modes and degrees of functional activity, and dogmatically state, with the facility of the old Yankee physician, "I understand your disaster; this is good for what ails you; you take this and you'll get well."

My regret is for the gratuitous statements made as principles involved in the strictures on the use of sterilized sponge to induce secondary dentine deposit over exposed pulps. I should be glad, had we the time at our disposal, to make some observations in this line myself. I am confident that an open quiz, orderly conducted, would bring out our strength of knowledge in such demonstrable shape as to relieve us from regrets for losing respect for the kind of statement, devoid of knowledge, referred to. Opportunity has not yet afforded me stimulus to prove campho-phenique. I have read and heard enough to predispose me to it.

Respecting the theory of the consolidation of amalgams (the perfect example of which is crystallization), I am wholly in accord with Dr. Elliott. This is too deep a question to be lightly referred to, and calls for a separate paper or brochure clearly to state what is already known, and to point the way to a better understanding of the subject.

All crystallization depends upon currents of energy manifested by separate points of polarization from which the crystals arise. The simplest example of this is the freezing of water on glass. No recommendation to dentists to study chemistry can be made too strong, from its absolute necessity to a professional education.

If any gentile present feels the inadequacy of my effort in form of statement or character of doctrine, I am ready to hear him and to reply to any question.

### REPORT OF SPECIAL OBITUARY COMMITTEE.

The committee to whom was referred the matter of the death of Robert V. Jenks, of Paterson, Frank S. Eggert, of Frenchtown, and Thomas L. Cook, of Long Branch, would most respectfully offer the following report:

WHEREAS, It has pleased the All-wise Ruler of the universe to remove from our midst our late companion and member, R. V. Jenks, of Paterson, Passaic County, N. J., while in the full practice of his profession, and at a good old age, it is fitting that we should place upon record a proper testimonial to his virtues. He was a regular attendant of our annual gatherings, and manifested a keen interest in all its proceedings. Diffident and retiring, rarely appearing in course of the debates during our meetings, yet to his friends in a private way he would show that he had a genius in the way of small inventions and mechanical contrivances.

*Resolved*, That while we deplore his loss to our Society we would express our sympathy for his family and immediate friends.

AND WHEREAS, Frank. S. Eggert, of Frenchtown, Hunterdon County, N. J., in the fifty-seventh year of his age, has gone from our midst into the higher life, we feel to condole our loss. He was one of the oldest members of our Society and, so far as any duty fell upon him, he was faithful in the performance of it; and among his townsmen he displayed the characteristics of a useful citizen.

*Resolved*, That we sympathize with his bereaved family and friends as we are sorry at the loss of a useful member of our Society.

AND WHEREAS, Thomas L. Cook, of Long Branch, Monmouth County, N. J., has departed this life and gone out of our midst, we trust to join a higher one in the mansions of eternal rest, depriving us of a useful member of our association.

*Resolved*, That while we mourn his loss, and would place upon the records our appreciation of his virtues, we would express our sympathy with the sorrowing family.

*Resolved*, That we place upon our minutes the above preambles and resolutions, and send a copy of the same respectively to their families, signed by the secretary under seal of the Society.

J. HAYHURST,<br>

C. S. STOCKTON, } *Committee.*<br>

B. F. LUCKEY,

*Dr. Hayhurst.*—Mr. President, I would like to say of Robert Jenks, that he was one of the five whom our President so feelingly referred to in his opening address. They were Drs. Chew, Cosad, Peirce, Jenks, and myself, men of advanced years, and we were mostly together. While the younger members of the Society preferred a more active manner of enjoyment, we associated ourselves together in a quiet, conversational way; and it always makes me think I am one step nearer that eternal rest which I have alluded to when it becomes my duty to speak of these bereavements. We are passing away, and when I look around and find that my associates in the beginning of this Society are becoming fewer and fewer every year, it is no wonder that sorrow comes into my heart and I feel that I am on the road to the beyond. You, young men, in the prime of life and the strength of manhood, who in the cultivation of your intellect enjoy advantages which we did not enjoy,—are you ready to take our places when we step from them? are you ready to uphold the dignity of the Society when you come among us? I hope you are; and if I could feel certain of it, as my connection with this Society nears its end, I would be happy and comfortable in that belief, and that the work which will descend upon your younger shoulders will be carried on as pleasantly, as socially, and with as much honor as when we older ones were in the harness. I thank you for your attention and for the quiet and impressive silence which seems to answer the appeal I have made.

### THE DENTAL PROTECTIVE ASSOCIATION.

The report of the Special Committee in the matter of the Dental Protective Association was read and adopted.

*Resolved,* That we heartily approve of the Dental Protective Association of the United States, and urge all who are not members to become so at once.

C. S. Stockton,<br>

W. Pinney, } *Committee.*<br>

Frank. M. Odell,

*Dr. Peirce.*—Mr. President, I want to say a word in regard to the Dental Protective Association. The matter is intensely interesting to me, and it seems to me that every member of this Society, as well as every member of the profession, owes it to himself and to his professional brethren to support that Association. I think I am familiar with its conception, and with the work that has been done, and I know there has never been an organization established within our order that bids fair to be of greater benefit to our profession at large than this Dental Protective Association that has been established in Chicago and of which Dr. Crouse is the chairman. I hope every member of this Society will feel it incumbent upon him to connect himself with it and forward his ten dollars for membership. You know we are periodically harassed by threats of suits, against which no individual feels able to defend himself; and here is an Association which says that for a paltry sum it will take these suits upon its shoulders and guarantee to protect and defend you against any combination that may come forward for the purpose of exacting illegal royalties.

*Dr. Sudduth.*—Mr. President, I have been, during the past year, in attendance upon the meetings in various States throughout the country, from the West to the extreme East, and have been surprised at the lack of interest in this movement, and, really, fears have been raised in my mind that it will fail on account of lack of interest on the part of the dental profession. In my opinion, it is the most vital question that has been brought forward in dentistry in the last decade. We must, as a profession, take hold and support this movement, or else we are going to have saddled on us a burden that will take twenty or twenty-five years to throw off. A gentleman, who is intimately connected with dental manufacturing, said to me the other day that he saw there was a possibility of its failure, through lack of interest and support on the part of the profession. If it does go down, you will have a repetition of the bull-dozing claims of the old Dental Vulcanite Company. It will only cost you ten dollars to get helped out of this difficulty now; it will cost you many times that amount if you do not combine for defence against these patents. The committee have about decided to adopt a limit to the number that shall be known as incorporators, who will come in by a payment of ten dollars; and you will wake up some day to find that it will cost you twenty-five dollars to get in. So you had better take the chance while you have it to get in for the smaller sum. Dr. Crouse made a proposition at Boston to the effect that any man who put his name down, with the intention of paying, would be considered a member, and given an opportunity to pay up at a later date, say some time the first of the year. If a suit is brought against you, the Association will not defend you unless you are a member; you must come in before a suit or injunction is brought in order to be defended by the Association.

(To be continued.)

